# Epithelial polarity and spindle orientation: intersecting pathways

**DOI:** 10.1098/rstb.2013.0291

**Published:** 2013-11-05

**Authors:** Dan T. Bergstralh, Timm Haack, Daniel St Johnston

**Affiliations:** The Gurdon Institute and the Department of Genetics, University of Cambridge, Tennis Court Road, Cambridge CB2 1QN, UK

**Keywords:** epithelial polarity, mitosis, spindle orientation

## Abstract

During asymmetric stem cell divisions, the mitotic spindle must be correctly oriented and positioned with respect to the axis of cell polarity to ensure that cell fate determinants are appropriately segregated into only one daughter cell. By contrast, epithelial cells divide symmetrically and orient their mitotic spindles perpendicular to the main apical–basal polarity axis, so that both daughter cells remain within the epithelium. Work in the past 20 years has defined a core ternary complex consisting of Pins, Mud and Gαi that participates in spindle orientation in both asymmetric and symmetric divisions. As additional factors that interact with this complex continue to be identified, a theme has emerged: there is substantial overlap between the mechanisms that orient the spindle and those that establish and maintain apical–basal polarity in epithelial cells. In this review, we examine several factors implicated in both processes, namely Canoe, Bazooka, aPKC and Discs large, and consider the implications of this work on how the spindle is oriented during epithelial cell divisions.

## 1. Introduction

Spindle orientation is important in both symmetrically and asymmetrically dividing cells. In asymmetrically dividing cells, the mitotic spindle needs to align parallel to the polarity axis, so that basal cell fate determinants segregate into only one of the two daughter cells, ensuring that this cell adopts a different fate from its sister. Many epithelial cells divide symmetrically. In these cells, the spindle is typically oriented in the plane of the tissue. This is thought to be important for the maintenance of epithelial integrity, because misoriented divisions will give rise to daughter cells that lie above or below the epithelial layer. A long-standing, albeit controversial, hypothesis suggests that continued division of such ‘extra-epithelial’ cells, isolated from their environment, could promote hypertrophy or tumour formation.

### (a) The Pins/Mud/Gαi complex

**Figure 1.**
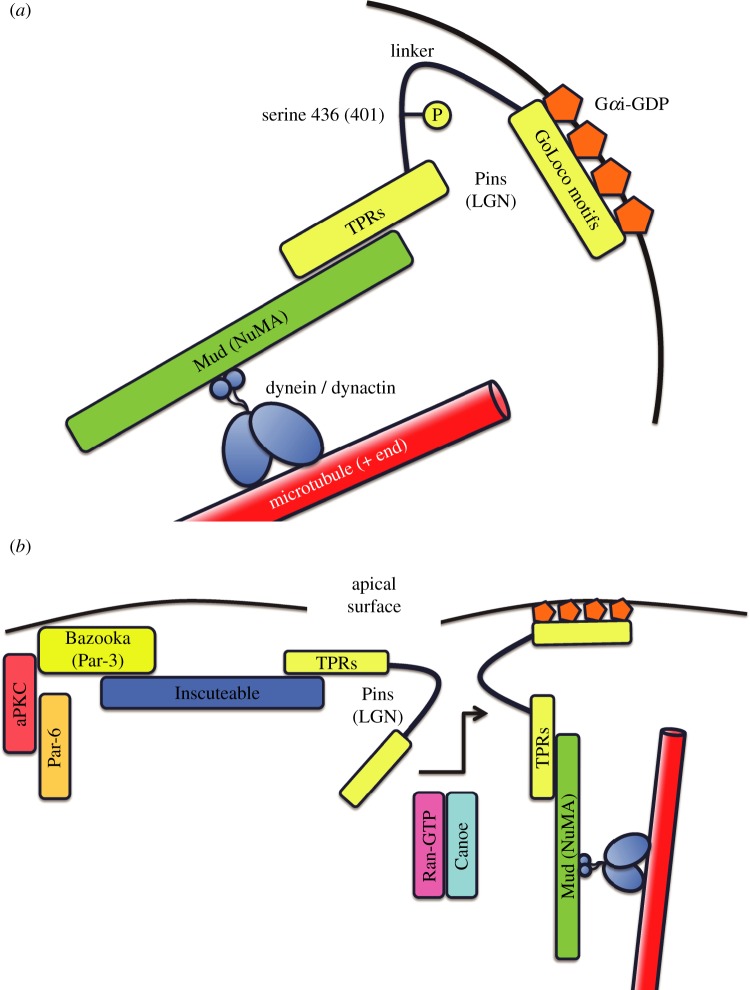
(*a*) A tripartite complex for spindle orientation. Mud (NuMA), Pins (LGN) and Gαi-GDP form a complex to capture astral microtubules. Mud binds to a dynein/dynactin complex that pulls on microtubule plus-ends to orient the spindle. The scaffold protein Pins provides the link between dynein/dynactin and the plasma membrane by binding to Mud via its N-terminal TPRs and to membrane bound Gαi-GDP via its C-terminal GoLoco motifs. Phosphorylation of serine 436 (S401 in humans) within the unstructured linker domain of Pins is required for spindle positioning. (*b*) Spindle orientation in neuroblasts relies on Pins recruitment via Inscuteable. Inscuteable apical localization requires Bazooka (Par-3), a component of the apical Par-6/aPKC/Bazooka complex. Inscuteable recruits Pins, but is replaced subsequently by Mud which also binds to the Pins N-terminal TPRs. Canoe, in cooperation with Ran-GTP, also binds to the TPRs to help recruit Mud. This specific interaction is not shown as the timing is unclear. Pins C-terminal domain binding to Gαi facilitates membrane anchoring of the Pins/Mud/Gαi tripartite complex and spindle orientation.

Studies in model organisms and mammalian cells have identified a conserved tripartite complex that plays a key role in spindle positioning in both asymmetric and symmetric cell divisions by recruiting dynein to the cell cortex, where it can capture astral microtubules to pull the spindle into alignment (figure 1*a*; recently reviewed in references [1,2]). The *Drosophila* complex consists of Partner of Inscuteable (Pins), Mushroom body defective (Mud) and the heterotrimeric G-protein subunit Gαi. As our review gives particular emphasis to *Drosophila*, these names will be given primacy through the article.

Pins was first recognized as a factor required for the asymmetric cell division of *Drosophila* neuroblasts [3]. It has since been shown to participate in mitotic spindle orientation in multiple tissues and organisms [2]. The vertebrate orthologue, Leu-Gly-Asn repeat-enriched protein (LGN), was identified as a spindle-associated factor and subsequently observed along the cortex of dividing cultured cells [4,5]. It plays a similar role in spindle orientation in vertebrate epithelial cells [1]. Two nearly identical Pins orthologues, G-Protein Regulators 1 and 2 (henceforth referred to as GPR-1/2), function redundantly to regulate spindle positioning in the *Caenorhabditis elegans* zygote [6–8].

Pins appears to act as a molecular scaffold for the spindle orientation machinery. Its N-terminal domain is composed of seven tetratricopeptide repeats (TPRs), which bind to Mud [4,9,10], and its C-terminal domain contains three GoLoco motifs that interact with guanosine diphosphate (GDP)-bound Gαi at the plasma membrane and function as GDP-dissociation inhibitors [5,11,12]. The TPRs and GoLoco motifs, which are connected by an unstructured linker domain, interact to hold the protein in an inactive conformation that is released upon binding of Pins to Mud and Gαi [12,13].

LIN-5 (abnormal cell lineage-5) was identified as a cell replication factor in *C. elegans* and subsequently shown to be required for the positioning of the spindle in the asymmetric division of the one cell zygote [14,15]. Its *Drosophila* orthologue, Mud, was recognized as a key factor in neuroblast spindle orientation [9,10,16]. The mammalian Mud orthologue Nuclear Mitotic Apparatus (NuMA) was first shown to bind LGN in a yeast two-hybrid screen using a library generated from mouse embryonic cells [4]. Similar to Pins/LGN, Mud is observed both at the spindle (where it recruits Pins) and at the cortex of dividing cells. Mud is a dynein binding protein, and exercises its effect on spindle orientation through the dynein/dynactin complex; this complex is required for spindle rocking and orientation in the *C. elegans* zygote and *Drosophila* larval neuroblasts, and cortical dynein/dynactin has been shown to act downstream of Gαi and LGN in spindle positioning in mammalian tissue culture cells [4,17–19].

Gαi-GDP, which binds to the plasma membrane via a myristoyl group, serves as a membrane anchor for Pins. Pins localization and spindle orientation in embryonic neuroblasts require Gαi, which is basolateral in epithelial cells, but localizes to the stalk and apical membrane in neuroblasts [20,21]. Gαi was initially identified as a spindle orientation factor in *C. elegans,* and has two orthologues (GOA-1 and GPA-16) [22]. Cortical localization of Gαi depends on the guanine exchange factor Ric-8, although its precise role is unclear [23–26]*.* A role for the G-protein subunit Gβγ in spindle orientation, probably as an antagonist to Gαi, is also suggested by several studies [22,25,27].

### (b) Epithelial polarity factors

**Figure 2.**
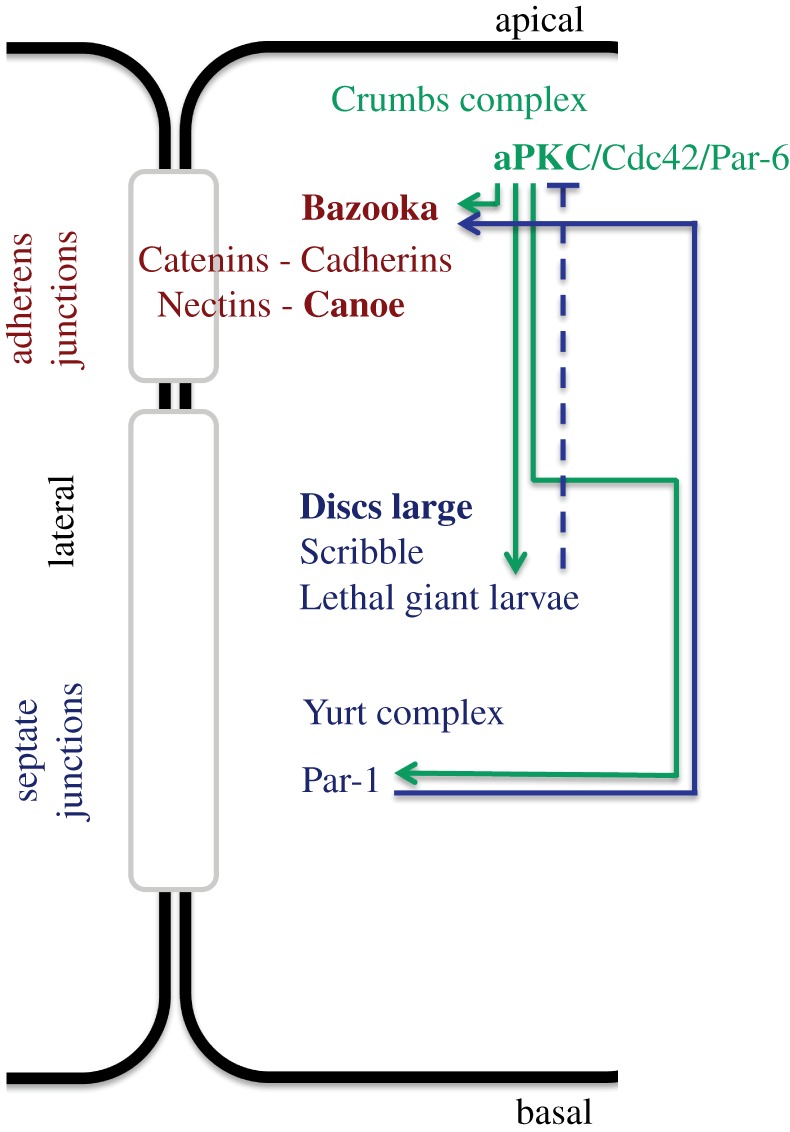
Epithelial polarity. Factors with roles in spindle orientation are highlighted in bold. Apicobasal polarity in *Drosophila* is determined by a set of conserved factors localizing to distinct domains. The apical domain is defined by the Crumbs complex and the Par-6/aPKC module which is regulated by Cdc42. Bazooka (mammalian Par-3) and Canoe (mammalian AF-6, Afadin) localize apicolaterally and regulate adherens junctions. The lateral domain is defined by a complex composed of Discs Large, Scribble and Lethal (2) giant larvae (Lgl), the Yurt complex and Par-1. Septate junctions are positioned below the adherens junctions in *Drosophila*. Mutual antagonism between apical and lateral factors, such as aPKC and Lgl, maintains apicobasal polarization. Arrows indicate the direct connections between some of these factors. A solid line indicates phosphorylation, whereas the dashed line indicates an undetermined inhibitory interaction.

Epithelial cells are defined by their apical–basal polarity, which is driven by factors acting in mutual opposition at distinct apical, junctional/lateral and basal cortical domains (figure 2) [28]. Ongoing work in multiple-cell types demonstrates that certain factors involved in determining epithelial polarity, namely Canoe (vertebrate AF-6 and Afadin), Bazooka (Par-3 in other organisms), aPKC and Discs large (Dlg), also participate in spindle orientation.

The first three of these factors can function together in epithelial cells. The kinase aPKC localizes along the apical cortex, where it acts in collaboration with other factors to regulate polarity. It is encoded by one gene in *Drosophila* but has two orthologues in humans: PKCι and PKCζ. (The latter is considered to be the functional equivalent of *Drosophila* aPKC, and for the sake of simplicity we will refer to it as aPKC.) Important targets for aPKC include Lethal giant larvae (Lgl), which is restricted to the lateral cortex by phosphorylation, and, in turn, restricts the localization of aPKC itself [29].

aPKC also phosphorylates Bazooka to position the apicolateral junctions [30–32]. It should be noted that the identity of these junctions differs between insect and vertebrate cells. In most *Drosophila* epithelia, adherens junctions are apicolateral and septate junctions basolateral (figure 2). This orientation is inverted in vertebrate cells; adherens junctions are basolateral, and tight junctions (the vertebrate equivalent of septate junctions) are apicolateral. However, the role of aPKC and Bazooka in positioning the more apical of the two junctions appears to be conserved [29]. Recent work shows that these proteins form a coregulatory protein network with a third factor, the PDZ protein Canoe, in initiating the polarity of new epithelial cells in the *Drosophila* embryo. Each protein is required for the correct localization and function of the other two [33].

A conserved genetic complex composed of three genes, *scribble*, *Discs large* (*Dlg*), and *Lethal* (*2*) *giant larvae* (*Lgl*), acts to define septate/tight junctions (figure 2) [34]. Dlg function is well characterized in *Drosophila* epithelial tissue, in which it is responsible for maintaining junctional integrity and epithelial polarization. It is more difficult to study in vertebrates. The most closely related mammalian protein is PSD-95, which also localizes laterally, but is one of four DLG orthologues that are thought to have some redundant functions.

In this review, we discuss the roles of these factors to spindle positioning in several organisms and cell types, beginning with the *C. elegans* embryo.

## 2. Asymmetric spindle positioning in *C. elegans*

Much of our understanding of both cortical polarity and spindle orientation is derived from studies performed in the single-cell *C. elegans* zygote. Upon fertilization, the zygote divides asymmetrically to generate a larger anterior AB cell and a smaller posterior P1 cell. Gradients set up prior to division ensure that certain determinants, including MEX-5 and MEX-6 (muscle excess 5/6) are inherited by the AB cell, which will go on to generate somatic tissues, whereas others are inherited by the P1 cell, which will generate germline and somatic cells [35].

Cortical polarity factors drive both the asymmetric distribution of cell fate determinants and the asymmetric positioning of the mitotic spindle, which is closer to the posterior cortex than to the anterior at division. Positioning is a two-step process. First, the maternal and paternal pronuclei, along with their centrosomes, migrate to the cell centre and the mitotic spindle forms along the anterior–posterior axis of the zygote. The spindle subsequently shifts towards the posterior cortex to make the division asymmetric (recently reviewed by Morin & Bellaïche [2] and Noatynska & Gotta [35]). Elegant laser-severing studies published in the early 2000s demonstrated that spindle movement during positioning relies on pulling forces, which are stronger at the posterior than at the anterior [8,36,37]. These forces, in turn, depend on the ternary complex and dynein [8,24,37,38]. Recent work helps to confirm that pulling occurs between the spindle and the cortex [39]. Depletion of non-muscle myosin promotes membrane invaginations during mitosis [39]. These invaginations are likely to be caused by weakening of the actomyosin cortex, so that the plasma membrane is pulled towards the spindle rather than vice versa. The polarized cortex thus plays a critical role in spindle positioning.

Genetic screening in the 1980s identified a set of *par* (partioning defective) genes required for asymmetric division of the *C. elegans* zygote [40]. Subsequent work in other organisms demonstrated that all PARs are highly conserved in animals with the exception of PAR-2 and that these genes regulate cortical polarity in multiple-cell types (reviewed by Goldstein & Macara [41]). In the *C. elegans* zygote, PAR-1 and PAR-2 localize at the posterior of the cell, where they act in mutual opposition to the anterior Par complex—PAR-3, PAR-6 and PKC-3 (the nematode orthologue of aPKC)—at the anterior cortex. The posterior and anterior proteins also determine the localization of another cortical factor, LET-99 (lethal-99), which localizes between them in a band along the lateral-posterior cortex [42,43]. These factors, in turn, determine the origin of pulling forces.

While GOA-1 and GPA-16 are uniformly distributed around the cortex, GPR-1/2 and LIN-5 show dynamic distributions throughout the cell cycle, coordinating with the pulling required for spindle positioning. Prior to metaphase, GPR-1/2 and LIN-5 are enriched at the anterior cortex, where they act to orient centrosomes along the anterior–posterior axis [44]. At metaphase, GPR-1/2 and LIN-5 redistribute to the posterior cortex, so that a stronger pulling force will bring the spindle further from the anterior, thereby causing asymmetry (reviewed by Morin & Bellaïche [2] and Noatynska & Gotta [35]). Along the lateral-posterior cortex, LET-99 inhibits pulling by preventing cortical localization of GPR-1/2 [45].

Recent work in *C. elegans* has also revealed a role for the polarity factor PKC-3 in regulating spindle position. PKC-3 is positioned along the anterior cortex of the nematode embryo and is required for asymmetric cell division [46]. Pronuclei ‘overcentre’ in *pkc-3* RNAi embryos, migrating past the cell centre to the anterior [46]. The relevant target for PKC-3 is LIN-5, which is phosphorylated along the anterior cortex in a cell-cycle- and PKC-3-dependent manner. Migration of the pronuclei from the posterior is slower in *lin-5* RNAi embryos, suggesting that dynein-pulling from the anterior is diminished. Rescue of this phenotype by expression of the non-phosphorylatable LIN-5 4A mutant promotes the same overcentering of the pronuclei observed after *pkc-3* RNAi. Thus, PKC-3 is thought to limit but not completely block LIN-5 activity [46]. Asymmetric cell division still occurs normally in LIN-5 4A *lin-5* RNAi cells, however [46]. This work demonstrates an intriguing connection between cortical polarity and the ternary complex, but explains only some aspects of how the PAR proteins regulate spindle positioning.

## 3. Asymmetric and polarized spindle orientation in *Drosophila*

The majority of spindle orientation studies in *Drosophila* have examined cells that divide in an asymmetric or polarized manner, such as neuroblasts and sensory organ precursor (pI) cells. A cleverly engineered cultured cell system with artificial polarity has also been used.

Studies in *Drosophila* neuroblasts have led to important insights into how cortical polarity and spindle orientation are coordinated to ensure the asymmetric outcome of the division: one self-renewing neuroblast and one ganglion mother cell (GMC), which divides once to produce two neurons or glial cells. As in epithelial cells, a complex of Bazooka (PAR-3), aPKC, Par-6 and Cdc42 defines apical–basal cortical polarity in neuroblasts by forming an apical domain that is limited, at least, in part, by antagonism with basal Lgl. The GMC fate of the basal daughter of the division is specified by a number of cell fate determinants, including the homeodomain transcription factor Prospero (Pros) [47–53], the tumour suppressor Brain tumour (Brat) [54–56] and the Notch repressor Numb [57–61]. Prospero and Brat both bind to the adapter protein, Miranda, which targets them to the basal cortex [62,63]. All three determinants are restricted to the basal cortex by aPKC, which phosphorylates Numb and Miranda to exclude them from the apical cortex [64,65]. Upon neuroblast division, the apical PAR proteins are inherited by the self-renewing neuroblast, whereas the basal factors are inherited by the GMC (reviewed by Egger *et al.* [66]).

Unlike epithelial cells, neuroblasts express the SH3 domain protein Inscuteable, which localizes apically and is required for proper spindle orientation and for the localization of basal factors [67]. Through interaction with the apical aPKC/Par-6/Bazooka complex (to which it binds directly), Inscuteable localizes along the apical cortex [68,69]. Inscuteable, in turn, recruits Pins to the apical membrane to promote spindle orientation along the apical–basal axis (figure 1*b*). Misexpression of Inscuteable in the embryonic ectoderm or in Madin–Darby canine kidney (MDCK) cells is sufficient to induce the ectopic apical recruitment of Pins, misorientation of the mitotic spindle and aberrant divisions perpendicular to the epithelial plane [20,67,70].

A suite of articles published in 2011 revealed the structural basis for the binding of Pins (LGN) to Inscuteable and to Mud (NuMA). Intriguingly, the binding sites overlap and are therefore exclusive; Pins can bind via its TPRs to either Inscuteable or Mud but not to both simultaneously [70–72]. This work suggests that although Inscuteable recruits Pins to the membrane, it must hand it off to Mud once there. This does not necessarily displace Pins from the apical cortex, as Pins is able to remain cortical through its interaction with Gαi.

### (a) aPKC is an important component of neuroblast polarity

Embryonic neuroblasts arise from the neuroectoderm by delamination and initially inherit the apical complex Bazooka/Par-6/aPKC from the epithelial cell layer. aPKC requires the small G-protein Cdc42 for apical localization and activation [65]. Expression of dominant negative Cdc42^DN^ in embryonic neuroblasts causes mislocalization of Par-6/aPKC, but Bazooka is still localized apically. In addition, *cdc42-3* mutant central brain neuroblasts lose aPKC and Par-6 from the apical cortex, whereas Bazooka stays localized. Furthermore, in the neuroblasts of zygotic *baz^4^* mutant embryos, Cdc42 apical enrichment is lost, and aPKC is mislocalized in the cytoplasm. This indicates that Bazooka acts upstream of aPKC in neuroblasts. Par-6 was reported to act as aPKC inhibitor [73–75], and Cdc42 was proposed to activate aPKC through lowering the inhibitory effect of Par-6 [65]. However, a more recent study showed that aPKC resides in an autoinhibitory state and is activated rather than inhibited by Par-6 binding [76].

The analysis of aPKC protein function in embryonic neuroblasts is hampered by the fact that aPKC is already required for epithelial polarity in the embryonic ectoderm, making it difficult to distinguish cell autonomous effects from general disruption of the epithelium in maternal/zygotic mutants. Larval central brain neuroblasts are derived from embryonic neuroblasts and are thus not formed directly from a polarized neuroepithelium. *aPKC^k06403^* zygotic mutants survive until late second-instar larval stage, allowing the analysis of larval neuroblast polarity [77]. In *aPKC^k06403^* mutants, Par-6 and Lgl are delocalized and Miranda spreads into the apical domain, but Bazooka, Pins and Dlg all localize correctly and spindle orientation is normal with respect to the Bazooka–Inscuteable–Pins apical crescent. Thus, aPKC is required to localize the basal determinants, but not for spindle orientation in this context.

Another role of aPKC is to antagonize Lgl. aPKC phosphorylation of Lgl causes its release into the cytoplasm, thereby restricting Lgl to the basal cortex [78]. In turn, Lgl was reported to act as inhibitor of aPKC [65,73]. Overexpression of non-phosphorylatable Lgl3A disrupts aPKC apical domain formation and Lgl can inhibit aPKC kinase activity *in vitro* [65]. However, Lgl is an aPKC substrate, and the interaction of these two proteins is likely to be short-lived *in vivo* [76]. It is therefore unclear whether the association of Lgl with aPKC inhibits the latter's activity *in vivo*, or whether the formation of the Lgl/aPKC complex is merely a consequence of aPKC being in an inactive state, so that it binds Lgl as a substrate but does not phosphorylate it.

It is also unclear what the actual role of Lgl at the basal cortex is. Initially, Lgl was suggested to be directly involved in targeting cell fate determinants to the basal cortex [78–81]. However, through its interactions with aPKC, the defects seen in Lgl3A overexpression and *lgl* mutants could be an indirect effect of loss of aPKC function or basal mislocalization, respectively [65,82].

Another model proposed that Lgl acts as a cell-cycle-dependent inhibitor of aPKC activity by antagonizing the assembly of the Bazooka/Par-6/aPKC complex [73]. These studies in larval neuroblasts and sensory organ precursor cells revealed the serine/threonine kinase Aurora A as an upstream regulator of aPKC activity, thereby linking regulation of the cortical polarity axis with progression through the cell cycle.

### (b) Canoe

Like Bazooka, Canoe localizes to adherens junctions during interphase in neuroepithelial cells of the *Drosophila* embryo [83]. Both proteins are observed along the apical cortex of delaminated metaphase neuroblasts and both participate in mitotic spindle alignment [83]. However, their roles appear to be substantially different. Although Bazooka acts through Inscuteable to regulate Pins localization, the apical localization and function of Canoe in these cells is downstream of Pins. Intriguingly, it is also upstream of Mud localization [83]. Work in cultured cells has illuminated this relationship. Canoe binds directly to the N-terminal Pins TPRs, whereas in combination with the GTP-bound form of the small GTPase Ran, it acts to recruit Mud to Pins [84]. How this activity coordinates with the binding of Pins TPRs to Inscuteable remains to be explored.

### (c) Discs large and Khc73

Dlg is a member of the membrane associated guanylate kinase (MAGUK) family of proteins, which is defined by a shared architecture that includes PDZ, SH3 and guanylate kinase (GUK) domains. These proteins associate with the plasma membrane via intermolecular interactions mediated by their PDZ domains. MAGUKs are thought to switch between active and inactive conformations; they are proposed to be held inactive by an intramolecular interaction between the SH3 and the GUK domain, which is C-terminal in Dlg and most other family members. The GUK domain, roughly 180 amino acids in length, is not catalytically active in these proteins but binds to phosphorylated partner proteins [85].

Interaction between Dlg and Pins was first demonstrated in the pI cell of the *Drosophila* pupal thorax, which divides in a planar-polarized manner along the anterior–posterior axis of the tissue [86]. In the dividing pI cell, Dlg and Pins colocalize along an anterior cortical crescent, opposite to Bazooka and aPKC, which form a posterior crescent [86]. In neuroblasts, Dlg is recruited apically to participate along with Pins in orienting the spindle along the apical–basal axis [1].

In special circumstances, Dlg can work upstream of Pins. In *inscuteable* mutant neuroblasts, Pins, Gαi and Dlg still form cortical crescents, although at a lower frequency and not necessarily at the apical surface. These secondary crescents require Dlg and the presence of astral microtubules [87]. This Inscuteable-independent pathway also requires the plus end-directed kinesin-3-family motor protein, Khc73 (GAKIN, for guanylate kinase associated kinesin, also called Kif13b in vertebrates) [87]. Khc73 was found to be associated with the GUK domain of Dlg in T lymphocytes, and this is also the case in neuroblasts [87,88]. One explanation for the secondary crescents is that Khc73 carries Dlg to the ends of astral microtubules, where it attaches to the cortex and recruits Pins. In support of this view, Khc73 is observed at the plus-ends of microtubules in mitotic neuroblasts [87]. Furthermore, disruption of Khc73 function prevents both Dlg and Pins from localizing to secondary crescents [87]. However, the secondary crescents still form some of the time in *dlg^IP20^* mutants, which lack the C-terminal 43 residues (and thus a large part of the GUK domain) of Dlg [87]. These residues are also dispensable for the anterior recruitment of Pins in pI cells [86]. Thus, the complete GUK domain of Dlg is not required to recruit either Dlg or Pins to the cortex, suggesting that its interaction with Khc73 is not essential, or that loss of the final 43 residues does not abolish this interaction. Even though *dlg^IP20^* mutants can form secondary crescents of Pins, the spindles usually fail to orient towards the crescent, indicating that the Dlg GUK domain also functions downstream of Pins in spindle alignment.

This function for Dlg downstream of Pins in spindle orientation is illuminated by studies performed in *Drosophila* S2-cultured cells [11]. Proteins fused to the homophilic adhesion molecule Echinoid are targeted to regions of the cell membrane in contact with another Echinoid-expressing cell, resulting in an artificially induced, polarized protein localization. In this system, a degree of spindle orientation is conferred by localizing just the Pins linker domain (residues 399–466), an unstructured stretch that falls between the N-terminal TPRs and C-terminal GoLoco motifs [11]. This domain recruits endogenous Dlg, which is required for the orientation effect [11].

How might this work? One possibility is that the Dlg GUK recruits Khc73, which, in turn, binds astral microtubules, thereby orienting one spindle pole. Multiple observations support this model: (i) *Dlg^1P20^* mutant neuroblasts are unable to correctly orient spindles towards Pins crescents [87]; (ii) spindles fail to achieve a settled orientation in *Dlg^1P20^* mutant pI cells [86]; (iii) membrane localization of the Dlg GUK domain alone, which should be sufficient to recruit Khc73, promotes some spindle orientation [11]; and (iv) disruption of Khc73 function by RNAi or by expression of a dominant negative prevents spindle orientation by both the Pins linker domain and by the Dlg GUK domain [11].

### (d) Does Aurora A phosphorylate Pins to promote binding to Dlg?

Although the GUK domain is not required to recruit Pins, it does bind directly to the Pins linker domain, which includes serine 436, a conserved phosphorylation site (S401 in humans) [11]. Phosphorylation of Pins at S436 is required for correct spindle position in both the Echinoid/S2 cell system and in larval neuroblasts and promotes the interaction with the Dlg GUK domain [11]. Pins S436 is a direct target of the mitotic kinase Aurora A *in vitro*, and *aurora-A* RNAi prevents the spindle orienting activity of membrane-targeted Pins in the S2 cell system [11]. Furthermore, spindle orientation is rescued in *aurora-A* RNAi cells and *pins* mutant neuroblasts upon expression of a phosphomimetic form of Pins [11]. Cumulatively, these findings suggest that Aurora A phosphorylates Pins at S436, thereby promoting the binding of Pins linker domain to Dlg to mediate correct spindle orientation.

## 4. Symmetrically dividing epithelial cells

Symmetrically dividing epithelial cells also show stereotypical orientation of mitotic spindles, but in contrast to neuroblasts, the spindles orient parallel to the plane of the tissue rather than perpendicular to it. Although considerably less attention has been paid to spindle orientation in symmetrically dividing *Drosophila* cells, symmetric division has been studied in vertebrate epithelial tissue and in cultured cells, in particular MDCK cell cysts, which are grown in three-dimensional culture to provide a simplified epithelium.

### (a) Par-3/Bazooka

A role for Par-3 in spindle orientation has also been demonstrated in MDCK cells [89]. As in neuroblasts, Bazooka is required for appropriate localization of Pins (LGN) in these cells. This function cannot be mediated through Inscuteable, which is not expressed in MDCK cells. It is also not simply a consequence of lost apicobasal polarity; several polarity markers localize appropriately in these cells [89]. However, Par-3 is required for apical localization of aPKC during interphase. The mechanism whereby Par-3 regulates LGN positioning has thus been suggested to be a consequence of disrupted aPKC function during mitosis [89].

### (b) Does aPKC phosphorylate Pins to exclude it from the apical cortex?

In MDCK cell cysts, LGN is cytosolic during interphase. During mitosis, LGN becomes enriched along the lateral and basal cortex but is excluded from the apical cortex [89]. In chick neuroepithelial cells, LGN is apical during interphase but, as in MDCK cells, becomes basolateral during mitosis [90]. In both systems, basolateral localization is thought to allow for the capture of astral microtubules such that the spindle is aligned parallel to the epithelial monolayer. Consistent with this model, knockdown of LGN leads to spindle misorientation in both MDCK cells and chick neuroepithelium [89,90]. As epithelial cells lack Inscuteable, other factors must be involved in localizing Pins along the basolateral cortex.

One strong candidate for directly influencing mitotic Pins/LGN localization is aPKC, because the exclusion of LGN from the apical cortex in dividing MDCK cells is prevented by an aPKC pseudo-substrate inhibitor [89]. The target site for aPKC phosphorylation of LGN is S401, the conserved residue (S436 in *Drosophila* Pins) also implicated in mediating the binding of Pins to Dlg. Phosphorylation of a C-terminal fragment of LGN at S401 is increased in cells expressing constitutively active aPKC and decreased in the presence of an aPKC pseudo-substrate inhibitor [89].

How does apical exclusion occur? A fragment of LGN which has been mutated (S401A) so that it cannot be phosphorylated at S401 binds to the membrane anchor Gαi more efficiently than the unmutated fragment [89]. One possibility is that another protein binds S401-phosphorylated LGN to physically block Gαi from binding. Consistent with this suggestion, the wild-type C-terminal fragment of LGN co-immunoprecipitates with the phosphoserine binding protein 14-3-3, but the S401A fragment does not [89]. Furthermore, the pseudo-substrate inhibitor of aPKC also prevents binding between 14-3-3 and LGN [89]. Although phosphorylation of the LGN at S401 by Aurora A has not been directly examined, chemical inhibition of Aurora A activity does not affect 14-3-3 binding [89]. In combination with earlier data, these results lead to a model in which aPKC-mediated phosphorylation of LGN at the apical cortex promotes binding of 14-3-3 to prevent binding to Gαi. LGN is thus excluded from the apical surface [89].

In agreement with this model, decreased aPKC function is suggested to prevent apical exclusion of Pins during mitosis in the imaginal discs of *Drosophila* larvae [91]. However, another study performed in cultured neuroepithelial cells produced conflicting results. In these cells, LGN was not cytoplasmic during interphase but rather localized along the apical cortex. Neither chemical inhibition of aPKC nor the expression of constitutively active aPKC altered the relocalization of LGN to the lateral cortex at mitosis. Moreover, mutation of serine 401 to alanine did not prevent apical exclusion of LGN in dividing cells, although lateral localization appeared weaker [90]. These findings suggest that that aPKC-mediated phosphorylation of Pins may be cell type or organism specific.

### (c) How and why is Pins phosphorylated?

Phosphorylation at S436 is required for spindle orientation towards Pins both in an S2 cell system and in larval brain neuroblasts [11]. Similarly, phosphorylation of LGN at S401 is required for spindle orientation in MDCK cell cysts [89]. Although phosphorylation is clearly important to Pins activity, work in different systems has led to two contradictory models in which this site is phosphorylated by aPKC to inhibit Pins or by Aurora A to activate it.

One model proposes that LGN is phosphorylated by aPKC to promote binding to 14-3-3, thereby preventing Pins from binding Gαi. This binding would exclude LGN from the apical cortex during mitosis. This raises a number of issues. (i) Although LGN phosphorylation is diminished in the presence of an aPKC inhibitor, LGN has not been shown to be a target of aPKC *in vitro*. (ii) aPKC would have to remain apical during mitosis to mediate the proposed exclusion. While aPKC localizes along the apical cortex in interphase MDCK cells, its localization during mitosis is not yet established. The mitotic localization of Gαi is likewise unknown in these cells. (iii) Gαi binds to the GoLoco domains of Pins, which are C-terminal to the phosphorylation site bound by 14-3-3. This makes a competition for binding seem less likely. (iv) Finally, it is worth noting that PKC-3 (aPKC) mediates spindle positioning in the nematode in part through phosphorylation of Lin-5 (Mud/NuMA) [46]. To the best of our knowledge, this activity has not yet been investigated in other organisms.

Another model proposes that phosphorylation of Pins by Aurora A promotes binding to the Dlg GUK domain. In turn, Dlg and Khc73 capture astral microtubules to promote spindle orientation. This model also raises questions. (i) As discussed previously, Aurora A can act upstream of aPKC during mitosis. Thus, inhibition of Aurora A may in fact inhibit aPKC-mediated Pins phosphorylation. It must be noted, however, that this suggestion conflicts with the alternative model, as chemical inhibition of Aurora A does not prevent binding of 14-3-3 to Pins [89]. (ii) The highly structured Dlg GUK domain is thought to bind phosphorylated Pins (S436) but also to Khc73. For stearic reasons, it would seem difficult for both to occur simultaneously. Is binding sequential? (iii) In mutant neuroblasts lacking Inscuteable, Dlg acts upstream of Pins to recruit it to the cortex. Most evidence, however, indicates that Dlg acts downstream of Pins in polarized and asymmetrically dividing cells. Does this interaction have a role in epithelial cells, in which Dlg is already localized at the lateral cortex?

## 5. Further questions

As appropriate to the dynamic state of the field, our review of the literature invites more questions than conclusions. While the most obvious of these surround the how and why of Pins phosphorylation, others are at least as interesting. Canoe, for example, is as yet unstudied in epithelial cell division. Does it have a function, and if so, is this role distinct from the one it plays at adherens junctions? What happens to these junctions in cells that do not delaminate from the epithelial layer? Given that some epithelial polarity factors have emerged as spindle orientation factors, would it be useful to test the involvement of others?

Finally, we note that recent work from the Cheeseman laboratory has illuminated a fascinating aspect of spindle orientation in HeLa cells, which lack apical–basal polarity. In these cells, the cortical positioning of LGN and NuMA is negatively regulated by a gradient of Ran-GTP originating from the chromosomes themselves [18]. Furthermore, Plk1 at the centrosomes negatively regulates the association of dynein/dynactin with NuMA [18]. This seems to be spindle orientation in reverse; cues originating from the metaphase plate and spindle poles affect the position and activity of the canonical orientation machinery, rather than the other way around. Our review has focused on the influence that this machinery, in cooperation with epithelial polarity factors, exerts on mitotic spindles. However, it is also possible that this regulation occurs in the opposite direction in epithelial cells.
